# Effects of Premature Primary Tooth Loss on Midline Deviation and Asymmetric Molar Relationship in the Context of Orthodontic Treatment

**DOI:** 10.7759/cureus.42442

**Published:** 2023-07-25

**Authors:** Ankita Warkhandkar, Lamia Habib

**Affiliations:** 1 Pediatric Dentistry, Rutgers School of Dental Medicine, Newark, USA; 2 Pediatric Dentistry, University of Connecticut School of Medicine, Farmington, USA

**Keywords:** treatment planning, orthodontics, molar relationship, midline deviation, tooth loss

## Abstract

Aim: The aim of this retrospective pilot study was to investigate the effects of premature loss of primary teeth on molar relationships and midline discrepancies in young patients and explore its potential implications for dental treatments, including orthodontics.

Materials and methods: A total of 550 study models from individuals seeking orthodontic treatment were evaluated, and 175 models met the inclusion criteria of mixed dentition. Each study model was examined for midline discrepancies, asymmetric molar relationships, and the impact of net premature tooth loss on the midline shift. Four investigators analyzed the data, and the error was minimized to less than 5%.

Results: The study revealed that premature loss of primary first molars was significantly associated with the midline shift, while premature loss of canines and second molars did not show substantial associations with midline discrepancies. The concordance analysis between observed and expected molar classifications based on premature tooth loss indicated relatively low concordance for all three molar classes, with higher concordance observed in Class II molar relationships. Notably, premature loss of primary second molars showed higher concordance than premature loss of primary first molars.

Conclusion: This retrospective pilot study highlights the potential dental complications associated with premature loss of primary teeth. Premature loss of primary first molars was found to be significantly linked to the midline shift, while the premature loss of canines and second molars did not demonstrate significant midline discrepancies. The concordance analysis revealed limited agreement between observed and expected molar classifications based on premature tooth loss, with higher concordance observed in Class II molar relationships and primary second molars. Further research with larger prospective cohorts and digital model analyses is warranted to gain a better understanding of the impact of premature tooth loss on dental treatment outcomes. The findings underscore the importance of early detection and appropriate intervention to mitigate potential adverse effects on dental arch development and malocclusion.

## Introduction

Premature loss of primary teeth leads to loss of space within the dental arch which is most commonly associated with issues such as dental caries, trauma, congenital disorders, and arch length deficiencies [1]. Additional adverse outcomes include loss of space within the arch, ectopic eruption, impacted teeth, Angle class II and class III malocclusions, midline deviation, and asymmetric molar relationships [1].

The effect of premature primary tooth exfoliation on space loss and future need for orthodontic treatment has been extensively reported in the literature. There was a significantly increased frequency of orthodontic treatment among individuals with premature loss of one or more deciduous teeth [2]. Another study conducted by Kumari and Retnakumari showed the influence of space loss on permanent dentition after the premature exfoliation of primary molars [3]. Arch length reduction of the maxilla and mandible and an increase in the arch length at the canine region of the mandible occur due to premature exfoliation of the primary molars [4]. It is generally accepted that the premature loss of deciduous teeth is associated with the malocclusion of the permanent dentition [5-7]. In an epidemiologic study conducted in Denmark, children with premature loss of primary teeth had more than three times the frequency of orthodontic treatment than those who had not lost their deciduous teeth prematurely; the premature loss of any primary molar resulted in space reduction for the permanent teeth [8]. The risk for major malalignment is 74% when a child lost one or more primary first molars at six years of age or younger. Later loss of primary first molars had a less severe effect on the production of major malalignment [2]. The earlier the premature loss of deciduous teeth, the greater the likelihood of crowding [9-12]. It was found that early loss of primary teeth would result in an increased frequency of sagittal, vertical, and transversal malocclusions, such that the need for treatment would increase significantly by early extraction of primary teeth. Whereas extraction in the maxilla would tend to result in the need for extraction of permanent teeth, extraction in the mandible would often lead to the need for orthodontic treatment of longer duration [13].

There have been conflicting data regarding the effect of premature tooth loss on future midline deviation. In some studies, only a slight increase in midline deviation was found in premature extractions in both arches, whereas extraction in one jaw only did not significantly alter the midline deviation [13]. In comparison, other investigators found that unilateral extraction had no influence on the maxillary midline while it caused a statistically significant dental shift to the extraction side in the mandible [14]. Regardless of the affected arch, a midline shift due to tooth movement into the primary canine space could contribute to midline deviation, complicating future orthodontic treatment. Bilateral loss of primary canines in a crowded arch leads to the lingual tipping of the permanent incisors, with the consequent reduction in the arch perimeter and increase in overbite. When the loss of a primary canine is unilateral, tipping of the adjacent incisors occurs toward the space, resulting in midline deviation [15].

The consequence of extraction of primary teeth was dependent on the site of extraction. Extraction in the maxilla would often lead to a distal molar occlusion due to frequent unilateral extraction in the maxilla to the rise in the frequency of unilateral distal occlusion. Extraction in the mandible seems to be followed by a decrease in the distal molar relationship, which could be explained by a mesial migration or tipping of the mandibular molars (though not enough in the majority of cases for the establishment of mesial molar occlusion) [13]. With premature primary molar loss, the permanent molar(s) often undergo mesial drift, affecting the molar relationship. When the primary molar is lost unilaterally, the resulting asymmetric molar relationship then complicates future orthodontic treatment [13].

No investigators have specifically studied the influence of tooth loss on midline discrepancies and asymmetry of molar relationship, two major problems complicating orthodontic treatment. Thus, the purpose of this retrospective study was to determine the effect of premature loss of primary teeth on molar and midline discrepancies.

## Materials and methods

The retrospective pilot study was conducted at the Children’s Dental Center in the Department of Pediatric Dentistry at the University of California, Los Angeles School of Dentistry. A total of 550 study models present in the department for orthodontic evaluation were evaluated, out of which 175 met the inclusion criteria of being in mixed dentition, teeth lost prematurely due to caries. Exclusion criteria included full primary dentition, full permanent dentition, missing teeth, or partially erupted teeth.

Each study model was evaluated for the following two criteria: midline discrepancies and asymmetric molar relationships.

Midline discrepancies 

Midlines were considered coincident if the mandibular midline was within +/− 1 mm to the right or left of the maxillary midline. Any difference greater than 1 mm was considered a midline discrepancy.

The reference point for the directional changes in this survey was established by keeping the maxilla as the stable point. The changes in the midline were derived by orienting the mandibular midline in alignment with the maxillary midline thus indirectly with the maxillary frenum. The constructed plane was drawn on the mandible, and any changes or shifts in the midline were calculated using a Vernier caliper by four examiners. Distances were recorded from the maxilla to the mandibular midline keeping the maxillary frenum as the stable point always. If there is diastema, the measurements were estimated to be the midpoints of the mesial surfaces of the maxillary central incisors.

Asymmetric molar relationships 

Molar relationships were measured using Angle’s classification. A Vernier digital caliper was used to determine molar relationships. Distances were measured between the mesiobuccal cusps of the maxillary first permanent molar to the buccal groove of the mandibular first permanent molar. The left molar distance was subtracted from the right molar distance to yield an asymmetric molar relationship if the molar classification was the same, added together if the molar classification was different.

The data were measured by four investigators. A digital caliper was used to record all measurements. Each measurement was recorded by three different examiners among four investigators who are pediatric dentists. The average measurement was recorded as the final measurement for analysis. The paired T-test was used for statistical analysis. All the study models were of children in the age group of 7-10 years of age. The specific primary teeth that were prematurely lost were determined based on the age of the child (7-10 years) and the sequence of tooth eruption. 

In order to quantify tooth loss, grids were created to show the loss in each quadrant and give a net loss as a whole (Table 1). This grid analysis was done for the primary canines, first molars, and second molars of each patient. The top row is for the maxilla, and the bottom row is for the mandible. Separate grids were made for primary canines, first molars, and second molars. Each square represents either the canine, first molar, or second molar in that quadrant. A “1” indicates that the canine, first molar, or second molar was lost prematurely in that specific quadrant, and a “0” indicates the tooth was not lost prematurely. A loss on the right side is recorded as a positive, and loss on the left side is recorded as a negative. If there are teeth lost on both sides of the maxilla or mandible, the net for that arch is 0. If teeth on the same side are lost, then the net loss is either 1 or −1 because both the upper and lower shifts would be predicted to the same side. If a tooth on one side of the maxilla is lost and a tooth on the other side of the mandible is lost, then the net loss is either 2 or −2 because the losses are predicted to have an additive impact on the shifting of the dentition.

**Table 1:** Example scenarios of net tooth loss

	Right	Left
Maxilla	0	0
Mandible	0	0
	Net loss: 0	

	Right	Left
Maxilla	1	0
Mandible	0	0
	Net loss: 1	

	Right	Left
Maxilla	0	1
Mandible	0	0
	Net loss: −1	

	Right	Left
Maxilla	1	1
Mandible	0	0
	Net loss: 0	

	Right	Left
Maxilla	1	1
Mandible	0	1
	Net loss: −1	

	Right	Left
Maxilla	1	0
Mandible	0	1
	Net loss: 2	

	Right	Left
Maxilla	1	1
Mandible	1	1
	Net loss: 0	

## Results

In order to compare our findings with our hypotheses, expected results based on specific teeth loss were recorded (Table 2).

**Table 2:** Expected effects of premature tooth loss on the midline and molar classification Primary upper first molars-#B,#I; primary upper second molars- #A,#J; primary lower first molars- #L,#S; primary lower second molars-#K,#T

		Canines				Primary 1^st^ molar				Primary 2^nd^ molar		
		C	H	M	R	B	I	L	S	A	J	K
	Effect on Medline	Maxilla to right	Maxilla to left	Mandible to left	Mandible to right	Mandible to right	Mandible to left	Mandible to left	Mandible to right	No change	No change	No change
	Effect on molar	No change	No change	No change	No change	Right side toward class II	Left side toward class II	Left side toward class II	Right side toward class II	Right side toward class II	Left side toward class II	Left side toward class II
		Canines				Primary first molar				Primary 2^nd^ molar		
		C	H	M	R	B	I	L	S	A	J	K
C	Effect on Medline	X	No change	Maxilla to right, mandible to left	No upper lower difference	Maxilla to right	No change	Maxilla to right, mandible to left	No upper lower difference	No change	No change	No change
C	Effect on molar	X	No change	No change	No change	Right side toward class II	Left side toward class II	Left side toward class II	Right side toward class II	Right side toward class II	Left side toward class II	Left side toward class II
H	Effect on Medline	X	X	No upper lower difference	Maxilla to left mandible to right	No change	Maxilla to left	No upper lower difference	Maxilla to left mandible to right	Maxilla to right	Maxilla to right	Maxilla to right
H	Effect on molar	X	X	No change	No change	Right side toward class II	Left side toward class II	Left side toward class II	Right side toward class II	Right side toward class II	Left side toward class II	Left side toward class II
M	Effect on Medline	X		X	No change	Maxilla to right, mandible to left	No upper lower difference	Mandible to left	No change	Mandible to right	Mandible to right	Mandible to right
M	Effect on molar	X		X	No change	Right side toward class II	Left side toward class II	Left side toward class II	Right side toward class II	Right side toward class II	Left side toward class II	Left side toward class II
R	Effect on Medline	X			X	No upper left difference	Maxilla to left mandible to right	No change	Mandible to right	Maxilla to right	Maxilla to right	Maxilla to right
R	Effect on molar	X			X	Right side toward class II	Left side toward class II	Left side toward class II	Right side toward class II	No difference	No differences	Left toward class II Right toward class II
B	Effect on Medline	X				X	No change	Maxilla to right, mandible to left	No upper lower difference	Maxilla to left	Maxilla to left	Maxilla to left
B	Effect on molar	X				X	No difference	Right toward class II, Left toward class III	No change	No difference	Left side toward class II	No change
I	Effect on medline	X					X	No upper lower difference	Maxilla to left, mandible to right	Maxilla to left	Maxilla to left	Maxilla to left
I	Effect on molar	X					X	No change	Left toward class II, right toward class III	No difference	No difference	Left toward class II Right toward class II
L	Effect on medline	X						X	No change	Mandible to left	Mandible to left	Mandible to left
L	Effect on molar	X						X	No difference	Right toward class II Left toward class III	No change	Left side toward class III
S	Effect on medline	X							X	Mandible to right	Mandible to right	Mandible to right
S	Effect on molar	X							X	No change	Left toward class II Right toward class III	No difference
A	Effect on medline	X								X	No change	No change
A	Effect on molar	X								X	No difference	Right toward class II left toward class III
J	Effect on medline	X									X	No change
J	Effect on molar	X									X	No difference
K	Effect on medline	X										X
K	Effect on molar	X										X
T	Effect on medline	X										
T	Effect on molar	X										

Statistical analysis of tooth loss and midline shift

Premature net canine loss, net first molar loss, and net second molar loss were subjected to statistical analysis using paired T-tests to investigate their potential association with the midline shift in the sample (Table 3). Among the 114 individuals included in the study, most showed zero net loss of any teeth, with only 15 individuals exhibiting net canine loss, 21 with net first molar loss, and 13 with net second molar loss.

**Table 3:** Effect of net premature loss of canines, first molars, or second molars on maxillary or mandibular midline shift

Canine loss	n	Mean	St. Dev.	P-value
−2	1	3.35	NA	0.29
−1	6	−1.16	1.6	
0	99	0.22	1.69	
1	7	-0.57	1.81	
2	1	-2.37	NA	
First molar loss				
−2	0	0	NA	0.065
−1	12	−0.71	1.79	
0	93	0.14	1.72	
1	8	0.87	1.78	
2	1	0	NA	
Second molar loss				
−2	0	0	NA	0.86
−1	1	2.88	NA	
0	101	0.04	1.67	
1	10	0.76	2.19	
2	2	−1.16	1.64	

The paired T-test results revealed relatively high p-values for the net canine loss group (p=0.29) and the net second molar loss group (p=0.86), indicating no statistically significant association with the midline shift. However, in the case of the net first molar loss group, the calculated p-value was 0.065, which is below the conventional significance level of 0.05. While not statistically significant, the lower p-value for the net first molar loss group suggests a potential association with the midline shift. Further investigations with a larger sample size might be needed to obtain more definitive conclusions.

It is important to note that the use of paired T-tests allowed us to compare the changes in the midline shift within each individual, thereby reducing the impact of confounding factors and improving the accuracy of the analysis. Despite the lack of statistically significant findings for net canine loss and net second molar loss, the data provide valuable insights into the potential impact of premature tooth loss on midline discrepancies, particularly concerning the premature loss of primary first molars. Additional research with larger sample sizes and prospective study designs is warranted to further explore these associations and validate the findings.

Statistical analysis of tooth loss and molar classification

To perform this comparison, we used a paired T-test, which is a statistical test used to determine if there is a significant difference between two related groups. In our case, the related groups were the expected molar classifications (based on the premature tooth loss) and the observed molar classifications.

We created two separate tables (Tables 4, 5) to present the data for the right and left molar classifications, respectively. Each table displayed the expected molar classifications in rows and the observed molar classifications in columns. The cells in the table indicated the number of cases that fell into each specific combination of expected and observed molar classifications.

**Table 4:** Concordance of expected vs observed molar classifications on the right side

Right	Expected		
Observed	I	II	III
I	0	2	9
II	0	14	6
III	0	0	3
Concordance: 50%			

**Table 5:** Concordance of expected vs observed molar classifications on the left side

Left	Expected		
Observed	I	II	III
I	0	7	7
II	0	10	11
III	0	1	2
Concordance: 32%			

After compiling the data, we calculated the concordance values, which represent the percentage of agreement between the expected and observed molar classifications. A concordance value of 100% would indicate a perfect agreement, while lower values suggest less agreement between the two.

The results of the paired T-test indicated that the concordance value for the right molar classification was 50%, while for the left molar classification, it was 32%.

The concordance analysis was conducted to compare the expected and observed molar classifications based on premature tooth loss (Tables 6, 7). In Table 6, which represents premature loss of primary first molars, the concordance value was found to be 31%. This indicates a 31% agreement between the expected molar classifications and the observed molar classifications for patients who lost their primary first molars prematurely. Similarly, in Table 7, which represents premature loss of primary second molars, the concordance value was higher at 50%. This indicates a 50% agreement between the expected molar classifications and the observed molar classifications for patients who lost their primary second molars prematurely. The statistical analysis using the paired T-test suggests that this difference in concordance values between primary first molars and primary second molars is statistically significant (p < 0.05), indicating a stronger association between premature loss of primary second molars and observed molar classifications.

**Table 6:** Concordance of expected vs. observed molar classification for primary first molar loss

	Expected		
Observed	I	II	III
I	0	8	10
II	0	12	12
III	0	1	2
Concordance: 31%			

**Table 7:** Concordance of expected vs. observed molar classification for primary second molar loss

	Expected		
Observed	I	II	III
I	0	1	10
II	0	12	6
III	0	0	5
Concordance: 50%			

## Discussion

The present study aimed to investigate the effects of premature loss of primary teeth on molar relationships and midline discrepancies in young patients, with implications for dental treatments, including orthodontics. Our findings provide valuable insights into this relatively underexplored area of research and contribute to the ongoing discussions in the field of dentistry and orthodontics.

Based on previous research by Ngan et al., we initially expected to observe a significant midline shift toward the side of loss in patients with missing primary canines and a less significant shift in those with missing primary second molars [16]. However, our results defied these expectations, revealing that only premature loss of first molars was associated with a noticeable midline shift (p=0.065). It was previously found that space loss associated with premature exfoliation of primary teeth usually resulted in distal movement of anterior teeth, thus causing a shift in the midline [17]. This was especially true for unilateral loss of maxillary first molars. The data from our study are relevant because the effects of the premature loss of primary first molars in the literature remain controversial.

Our study sample comprised study models from individuals seeking orthodontic treatment, many of whom had contralateral or all four canines missing. Dental providers often extract the contralateral tooth in patients with unilateral canine loss to maintain midline symmetry before taking orthodontic records. Consequently, our study models may not accurately represent the effects of premature loss of canines, as midline discrepancies were addressed prior to creating the models.

As shown in previous research, mesial drift of permanent molars is a common phenomenon following the loss of teeth distal to an edentulous space [18,19]. We hypothesized a molar relationship shift toward Class III with premature loss of a mandibular primary posterior tooth and a shift toward Class II with premature loss of a maxillary primary posterior tooth. To analyze molar relationships, we recorded the observed molar classification of each study model and calculated the concordance correlation coefficient to assess the agreement between observed and expected molar classifications based on which tooth was exfoliated. However, a limitation was that we could not determine the patient's molar classification before the speculated tooth loss. Furthermore, we expected higher concordance for second molars than first molars in molar relationships, considering the faster and greater closure of the second molar extraction space compared to the first molar extraction space [2]. Concordance was relatively low for expected versus observed molar relationships in all three classes, with the highest concordance found among Class II molar relationships. Additionally, premature loss of primary second molars showed higher concordance than primary first molars. The research on asymmetric molar relationships is limited, making our study more challenging in this subject area.

Limitations

Retrospective Study Design

The retrospective nature of our study may have introduced biases, as we relied on existing records and models. There may have been inconsistencies in the data collection process, and selection bias could have influenced the sample composition.

Sample Size

The small sample size of patients with premature tooth loss limited the generalizability of our findings. A larger and more diverse sample would have provided more robust results.

Subgroups Within the Sample

Subgroups within the sample, presenting with diastema and/or contralateral tooth loss, further reduced data pertaining to midline shift. Future studies should consider accounting for these subgroups separately.

Prior Space Maintenance and Serial Extractions

The presence of prior space maintenance and serial extractions in many individuals may have skewed the data, preventing us from examining the isolated effects of tooth loss.

Speculation About Tooth Loss and Treatment Stage

Speculating about premature tooth loss and the stage of dental treatment for each patient was a limitation, as we could not access records to determine if space maintenance had been attempted.

To improve future research, a prospective cohort study design would eliminate the need for speculation and provide a more comprehensive understanding of the effects over time. Incorporating digital model analyses could also enhance measurement accuracy and minimize discrepancies in molar relationship assessments. Addressing these limitations would help advance knowledge in this underexplored area of research.

## Conclusions

In conclusion, this study provided valuable insights into the effects of premature tooth loss on molar relationships and midline discrepancies in young orthodontic patients. Contrary to initial expectations, only premature loss of primary first molars was associated with a noticeable midline shift. Premature loss of primary canines and second molars did not demonstrate significant midline changes. The concordance analysis showed relatively low agreement between expected and observed molar relationships, with higher concordance found among Class II molar relationships. Premature loss of primary second molars exhibited higher concordance compared to primary first molars.

However, the study faced limitations, including its retrospective design, small sample size, and subgroups with diastema and contralateral tooth loss. The impact of prior space maintenance and serial extractions in many patients may have influenced the isolated effects of tooth loss.

Despite these limitations, our research contributes to the understanding of the consequences of premature tooth loss in orthodontic treatment planning. Identifying specific tooth loss associated with midline shifts and molar relationship changes highlights the importance of considering individual tooth loss patterns.

Our study highlights the importance of personalized treatment strategies in orthodontic care, considering the effects of premature tooth loss on molar relationships and midline discrepancies. To advance research, we suggest prospective cohort studies and digital model analyses for better data collection. Further investigations are needed to validate and enhance our understanding of premature tooth loss and its impact on orthodontic treatment.
